# *GmWRKY40*, a member of the WRKY transcription factor genes identified from *Glycine max* L., enhanced the resistance to *Phytophthora sojae*

**DOI:** 10.1186/s12870-019-2132-0

**Published:** 2019-12-30

**Authors:** Xiaoxia Cui, Qiang Yan, Shuping Gan, Dong Xue, Haitang Wang, Han Xing, Jinming Zhao, Na Guo

**Affiliations:** National Center for Soybean Improvement, Key Laboratory of Biology and Genetics and Breeding for Soybean, Ministry of Agriculture, State Key Laboratory of Crop Genetics and Germplasm Enhancement, Nanjing Agricultural University, Nanjing, 210095 China

**Keywords:** *Glycine max* (L.) Merr, *Phytophthora sojae*, GmWRKY40, RNA interference, Yeast two-hybrid, Soybean hairy roots

## Abstract

**Background:**

The WRKY proteins are a superfamily of transcription factors and members play essential roles in the modulation of diverse physiological processes, such as growth, development, senescence and response to biotic and abiotic stresses. However, the biological roles of the majority of the WRKY family members remains poorly understood in soybean relative to the research progress in model plants.

**Results:**

In this study, we identified and characterized *GmWRKY40*, which is a group IIc *WRKY* gene. Transient expression analysis revealed that the GmWRKY40 protein is located in the nucleus of plant cells. Expression of *GmWRKY40* was strongly induced in soybean following infection with *Phytophthora sojae*, or treatment with methyl jasmonate, ethylene, salicylic acid, and abscisic acid. Furthermore, soybean hairy roots silencing *GmWRKY40* enhanced susceptibility to *P. sojae* infection compared with empty vector transgenic roots. Moreover, suppression of *GmWRKY40* decreased the accumulation of reactive oxygen species (ROS) and modified the expression of several oxidation-related genes. Yeast two-hybrid experiment combined with RNA-seq analysis showed that GmWRKY40 interacted with 8 JAZ proteins with or without the WRKY domain or zinc-finger domain of GmWRKY40, suggesting there were different interaction patterns among these interacted proteins.

**Conclusions:**

Collectively, these results suggests that *GmWRKY40* functions as a positive regulator in soybean plants response to *P. sojae* through modulating hydrogen peroxide accumulation and JA signaling pathway.

## Background

*Phytophthora* spp. are oomycetes that cause devastating diseases on a number of important crops. For example, the pathogen *Phytophthora infestans* was responsible for a devastating outbreak of potato blight in Ireland [1]. *Phytophthora sojae* is the second most destructive pathogen of soybean could led to Phytophthora root and stem rot (PRR), causing annual losses of approximately 1–2 billion US dollars globally [2]. *P. sojae* is widespread, occurring in all major soybean growing regions. PRR can cause total loss of the crop in highly susceptible cultivars. Disease resistance breeding is the main strategy to control oomycete diseases. Presently, PRR is mainly managed on the basis of host-mediated resistance provided by resistance (*R*) genes. In the soybean-*P. sojae* pathosystem, the types of resistance have been described, including race-specific resistance controlled by single dominant *Rps* (resistance to *P. sojae*) genes and partial resistance provided by multiple genes [3, 4]. In soybean, 33 *Rps* genes have been identified and mapped to eight chromosomes [5–9]. Among them, *Rps1a*, *Rps1c*, *Rps1k*, *Rps3a*, *Rps6*, and *Rps7* have successively been introgressed in commercial lines [10, 11]. However, resistance genes will be overcome after widespread and persistent infection of pathogens, therefore, their effectiveness is usually transient [12]. Partial resistance is different from *Rps*-mediated resistance, it is controlled by multiple genes located in multiple loci, and each gene contributing a moderate effect [13, 14]. It has been shown that partial resistance of soybean can effectively against a variety of pathotypes of *P. sojae* [15, 16]. Studies on the mechanism of this pathological system have provided evidence for the participation of *R* gene and constituent part of defense signal pathways [17–19]. Schneider et al. conducted GWA analysis to identify candidate genes and coincident QTLs to investigate the mechanism hypothesis of partial resistance, so as to provide evidence for different hypotheses such as weakened *R* gene response and some genes that participated in basal defense and signal pathways [20]. Using RNAi approach, soybean *GmSGT1* was identified positively contribute to partial resistance and are differently required for soybean *Rps1a*, *Rps1c*, *Rps1d*, *Rps1k*-mediated disease resistance [21]. Genetic engineering has the potential to confer durable and broad-spectrum resistance on plant by expressing antimicrobial components of known defense signaling pathways [22]. To date, researches have been conducted to increase soybean resistance to *P. sojae* infection by expressing foreign genes which confer resistance to fungal pathogens. Soybean *defender against apoptotic death 1* (*DAD1*), that might participate in the endoplasmic reticulum stress signaling pathway, plays a critical role in defense against *Phytophthora* pathogens [23]. A soybean novel BTB/POZ domain-containing nuclear protein GmBTB/POZ plays a positive role in the soybean response to *P. sojae* [24]. The basic helix-loop-helix (bHLH) transcription factor GmPIB1, which repress the expression of *GmSPOD1* by directly bind to its promoter, enhances the resistance of soybean to *P. sojae* [25]. Overexpression of ethylene response factor *GmERF5* and *GmERF113* increased the soybean resistance to *P. sojae* and positively regulated the expression of the pathogenesis-related genes [26, 27].

Transcriptional regulation of gene expression is mainly mediated by *trans*-acting sequence specific DNA binding TFs specific recognition *cis*-acting promoter elements [28, 29]. DNA binding proteins containing WRKY domains are one of several transcription factor families associated with plant defense responses [30, 31]. WRKY TFs presence of one or two conserved WRKY domains with about 60 amino acid, these domains followed by a Cys_2_His_2_ or Cys_2_HisCys zinc-finger motif [32]. In plant-pathogen interaction, WRKY TFs involved in the regulation of plant defense mechanisms (PTI and ETI) by recognizing and binding to the W-box or W-like box-type *cis* elements or other *cis* elements in promoter regions of some genes [33, 34]. Such as *Arabidopsis* WRKY18, WRKY33, and WRKY40 bind to W-box [TTGAC(C/T)] motif during early MAMP-triggered immunity [35]; OsWRKY51 bind to the *cis*-element W-box and WLE1 [(T)TGACA] on the promoter of the defense gene *OsPR10a*, enhances plant resistance to *Xoo* by activating the expression of this gene [36]. The first WRKY protein SPF1 was cloned from sweet potato in 1994, so far, a growing number of WRKY genes have been identified in various plant species, such as at least 72 members in *Arabidopsis thaliana* [31], 109 in rice [37] and 182 in soybean [38]. Loss-of-function and gain-of-function studies have demonstrated that WRKY transcription factors act in a complex signaling network as both positive and negative regulators of disease resistance [31, 39, 40]. A number of studies have shown that WRKY TFs have regulatory functions in plant defense responses to various pathogens infection. For example, in *Arabidopsis*, overexpression of *AtWRKY18* and *AtWRKY70* results in increased resistance to *Pseudomonas syringae* and enhanced expression of SA-responsive pathogenesis-related (PR) genes [39, 41]. Furthermore, *Arabidopsis* WRKY33 is essential for defense toward the *Botrytis cinerea*, and genes involved in SA signaling, ethylene-JA-mediated cross-communication and camalexin biosynthesis were identified as direct targets of AtWRKY33 [42]. In rice, overexpression of *OsWRKY45* led to enhanced resistance to *Magnaporthe grisea*, and *OsWRKY45* is proposed play a important role in Benzothiadiazole-induced and SA-mediated defense signaling in rice [43]. Overexpression of *OsWRKY6* in *Arabidopsis* exhibited increased disease resistance to *Xanthomonas oryzae* through the induction of defense-related genes such as *PR1*, *PDF1*, *NPR4* and *glucanase* [44]*.* Overexpression of pepper *CaWRKY40* in tobacco enhanced resistance to *Ralstonia solanacearum* and modified the expression of hypersensitive response (HR)-associated and pathogenesis-related genes [45]. Members of the genus *Phytophthora* are among the most serious threats to agriculture and food production, causing devastating diseases in hundreds of plant hosts [46]. Recently WRKY TFs participate in plant-*Phytophthora* interactions has been further studied. Adachi et al. identified that WRKY TFs phosphorylated by MAPK play important roles in RBOHB-dependent ROS burst and cell death-mediated immunity to *Phytophthora infestans* in *Nicotiana benthamiana* [47]. Potato *StWRKY8* positively regulate the *Arabidopsis* resistance to *P. infestans* by interacting with the promoters of benzylisoquinoline alkaloids biosynthetic genes [48]. In *Solanum pimpinellifolium,* Eight WRKY TFs were identified in *S. pimpinellifolium-P. infestans* interaction and transgenic tomato overexpressing *SpWRKY3* showed a significant resistance to *P. infestans* [49]. Overexpression of *SpWRKY1* in tobacco conferred resistance to *P. nicotianae* infection [50].

Soybean (*Glycine max* (L.) Merr.) is a leguminous crop with great economic and agricultural importance all over the world. To date, only a small number of WRKY TFs in soybean have been cloned and functionally characterized. The role of WRKY TFs in abiotic stress responses and growth and development in soybean has been identified. Overexpressing *GmWRKY21* or *GmWRKY54* enhances cold, salt and drought tolerance in *Arabidopsis thaliana*, by contrast, it is more sensitive to salt and mannitol stress while overexpressing *GmWRKY13* [51]. Virus-induced silencing of *GmWRKY58* or *GmWRKY76* resulted in a reduction of leaf expansion and termination of stem growth [52]. WRKY-related protein GmWRP1 not only plays an important roles in leguminous plant symbiosis, but also important in the growth and development of leguminous [53]. However, only a few have been characterized in biotic stress responses. It was reported that overexpression of *GmWRKY31* enhanced resistance to *P. sojae*, while silencing the same gene enhanced susceptibility [54]. In the present study, the function of *GmWRKY40*, a putative Group IIc WRKY gene, was characterized, and the gene expression patterns in response to different stress conditions were analyzed. Transgenic soybean hairy roots silencing *GmWRKY40* increased susceptibility to *P. sojae*. Moreover, lower levels of ROS was observed in *GmWRKY40*-RNA interference roots. Further analysis indicated that GmWRKY40 physically interacted with JAZ proteins. These results suggest that *GmWRKY40* functions in soybean response to *P. sojae* through modulating ROS accumulation or JA signaling pathways, and it is an essential component for soybean against *Phytophthora* pathogens.

## Results

### Identification and sequence analysis of GmWRKY40

**Fig. 1:**
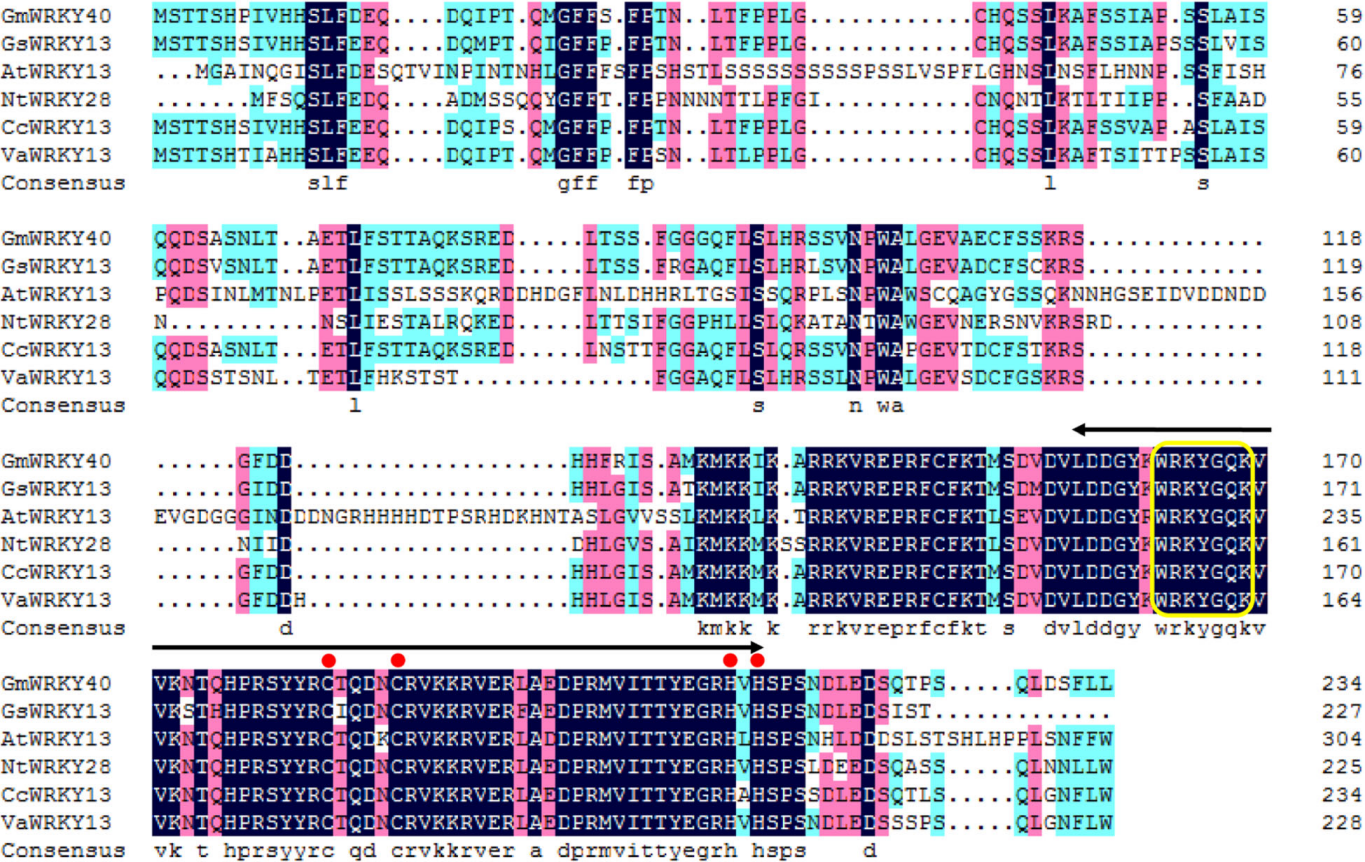
Characterization and sequence analysis of GmWRKY40. Sequence alignment of the deduced GmWRKY40 protein with *Glycine sojae* WRKY13 (KHN34020.1), *Cajanus cajan* WRKY13 (XP_020215926.1), *Vigna angularis* WRKY 13 (XP_017432586.1), *Nicotiana tabacum* WRKY 28 (XP_016452899.1) and *Arabidopsis thaliana* WRKY13 (NP_195651.1). The dark blue (100%), pink (80%), and cyan (60%) boxes represent levels of amino acid identity or similarity. The double arrow indicates the WRKY domain. The highly conserved amino acid sequence is boxed. The C and H residues in the zinc-finger motif are marked by red dots

In previous study, we screened for soybean genes that response to *P. sojae* infection by using RNA silencing in soybean cotyledons according to the method of Subramanian et al. [55] and Yan et al. [21]. Among these, silencing of *GmWRKY40* in soybean cotyledon displayed enhanced susceptibility to *P. sojae*. Moreover, *GmWRKY40* was up-regulated in the leaves of soybean following infection with *P. sojae* (Fig. 3a), and *GmWRKY40* was thus chosen for the further analysis of its role in response to *P. sojae*. *GmWRKY40* is located on chromosome 8, designated with the locus number Glyma.08G143400. *GmWRKY40* cDNA is 1296 bp, with a 708 bp open reading frame (ORF), encoding a protein with 235 amino acids. Protein structure analysis revealed that GmWRKY40 harbors an approximate 60-amino acid WRKY domain that is composed of the conserved amino acid sequence (WRKYGQK) and a zinc-finger motif (C-X_4–5_-C-X_22–23_-H-X_1_-H), indicating that GmWRKY40 belongs to Group IIc of the WRKY family [32]. BLASTp analysis revealed that the amino acid sequence of GmWRKY40 had an 90% sequence identity with the putative WRKY transcription factor 13 (KHN34020.1) from *Glycine sojae* and probable WRKY transcription factor 13 (XP_020215926.1) from *Cajanus cajan*, an 83% sequence identity with the probable WRKY transcription factor 13 (XP_017432586.1) from *Vigna angularis*, an 62% sequence identity with the probable WRKY transcription factor 28 (XP_016452899.1) from *Nicotiana tabacum* and an 49% sequence identity with the WRKY13 (NP_195651.1) from the *Arabidopsis thaliana* (Fig. 1). These results indicated that GmWRKY40 is highly conserved in legumes.

### Subcellular localization of GmWRKY40

**Fig. 2:**
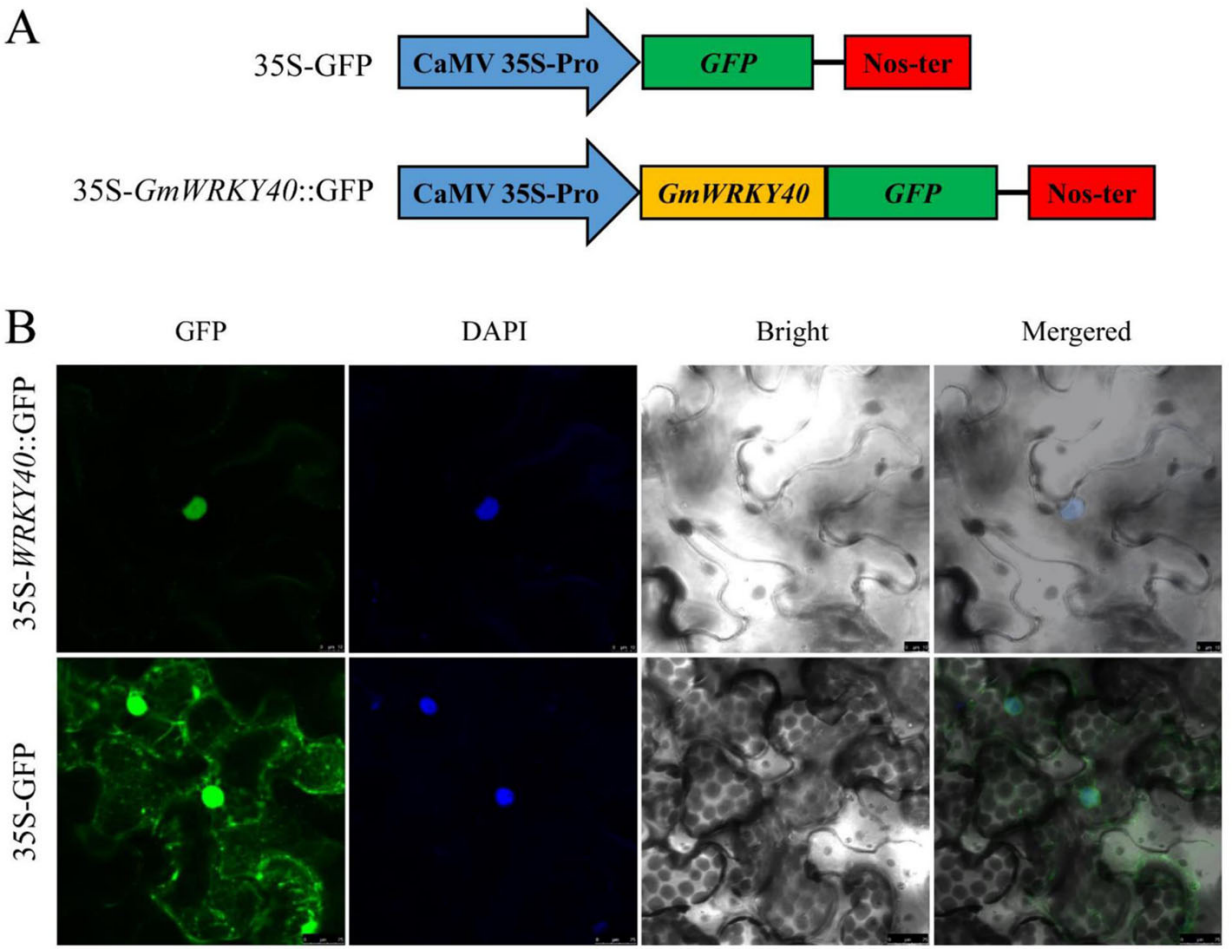
Subcellular localization of the GmWRKY40 protein. **a** Schematic diagram of the 35S-*GmWRKY40*::GFP fusion construct and the 35S-GFP construct. **b** Agrobacteria carrying 35S-*GmWRKY40*::GFP and 35S-GFP were infiltrated into leaves of *N. benthamiana* plants. Pictures were taken in bright and fluorescence fields after DAPI staining with confocal laser scanning microscopy 72 h after agroinfiltration

To confirm its nuclear localization, the construct was generated in which the full ORF of *GmWRKY40* was fused in frame to green fluorescent protein (GFP) under the control of the constitutive *CaMV 35S* promoter, and the *p35S-GFP* used as a negative control (Fig. 2a). The fluorescence was observed by confocal microscope with DAPI staining to detect the nuclei. Typical results indicated that the GFP fluorescence and DAPI staining were predominantly localized to the nucleus expressing *35S-GmWRKY40::GFP*, whereas the *35S-GFP* control exhibited the fluorescence in both the nuclei and the cytoplasm (Fig. 2b). These suggested that GmWRKY40 protein is localized in the nucleus, and this localization may help us to explore the function of this protein.

### Response of *GmWRKY40* transcript to *P. sojae* infection and various exogenous phytohormones

**Fig. 3:**
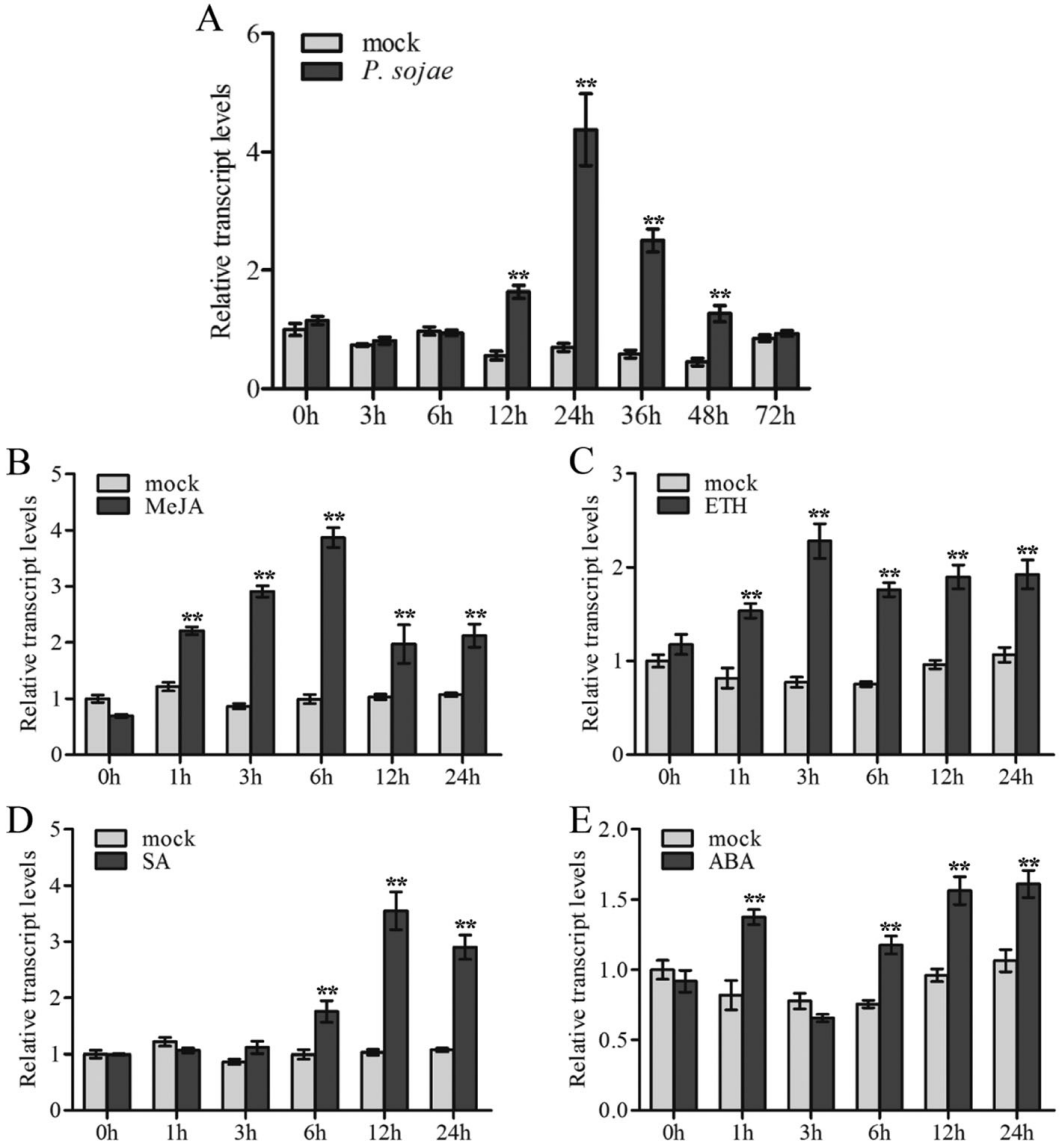
Expression patterns of *GmWRKY40* under diverse stress conditions. Approximately 1-week-old soybean seedlings were used for each of the following treatments. **a** The relative expression of *GmWRKY40* was determined at different time points in the soybean hypocotyl after inoculation with *P. sojae*. **b**-**e** *GmWRKY40* transcripts examined in soybean roots at various time periods after treatment with 100 μM MeJA, 2 mM ETH, 5 mM SA and 50 mM ABA. Error bars indicate the standard error; the experiments were repeated three times along with at least three independent repetitions of the biological experiments. Asterisks indicate statistically significant differences (^**^P < 0.01)

To examine the effects of *GmWRKY40* in response to pathogen defense, the transcript level of *GmWRKY40* in the leaves of soybean in response to infection with *P. sojae*, was determined by quantitative real-time PCR (Fig. 3a). Compared with the mock, *GmWRKY40* transcripts were up-regulated in leaves at 12–48 h post-inoculation with *P. sojae*, and with a peak at 24 h. The induction of *GmWRKY40* transcript suggested that *GmWRKY40* may play an important role in plant resistance to oomycete pathogens.

Phytohormones, such as methyl jasmonate (MeJA), ethylene (ETH), salicylic acid (SA) and abscisic acid (ABA), serve as significant signaling molecules in the regulation of plant defense responses to various biotic stresses and play important roles in mediating the expression of downstream defense-related genes [56]. To evaluate the possible roles of *GmWRKY40* in the signaling cascades utilized by these hormones, *GmWRKY40* transcript levels were monitored by qRT-PCR in 7-day-old soybean roots treated with MeJA, ETH, SA, and ABA. As shown in Fig. 3b-e, after the 100 μM MeJA treatment, *GmWRKY40* expression increased within 1 h to 24 h post-treatment, with a peak at 6 h (3.9-fold). In response to 2 mM of ethylene, the *GmWRKY40* transcript level was notably enhanced from 1 h to 24 h. The expression of *GmWRKY40* was obviously induced at 6 h after 5 mM SA treatment and approaching highest (3.4-fold compared with mock) levels at 12 h. Under 50 mM ABA treatment, the expression of *GmWRKY40* increased dramatically at 1 h and reduced to the mock-treated levels at 3 h, then highly induced around 6 h–24 h. These results indicated that *GmWRKY40* may be involved in pathogen responses through the plant hormones signaling pathways.

### Suppression of *GmWRKY40* increased soybean susceptibility to *P. sojae*

In order to evaluate the biological function of *GmWRKY40*, *GmWRKY40* was silenced in soybean hairy roots to tests the effect on resistance. The hairy roots with GFP fluorescence were isolated for assay (Additional file 1: Figure S1A). To confirm the silencing efficiency, we performed quantitative real-time PCR, which showed a significant decrease in the RNAi-*GmWRKY40* fluorescent roots compared to the RNAi empty vector control roots (Additional file 1: Figure S1B).

**Fig. 4:**
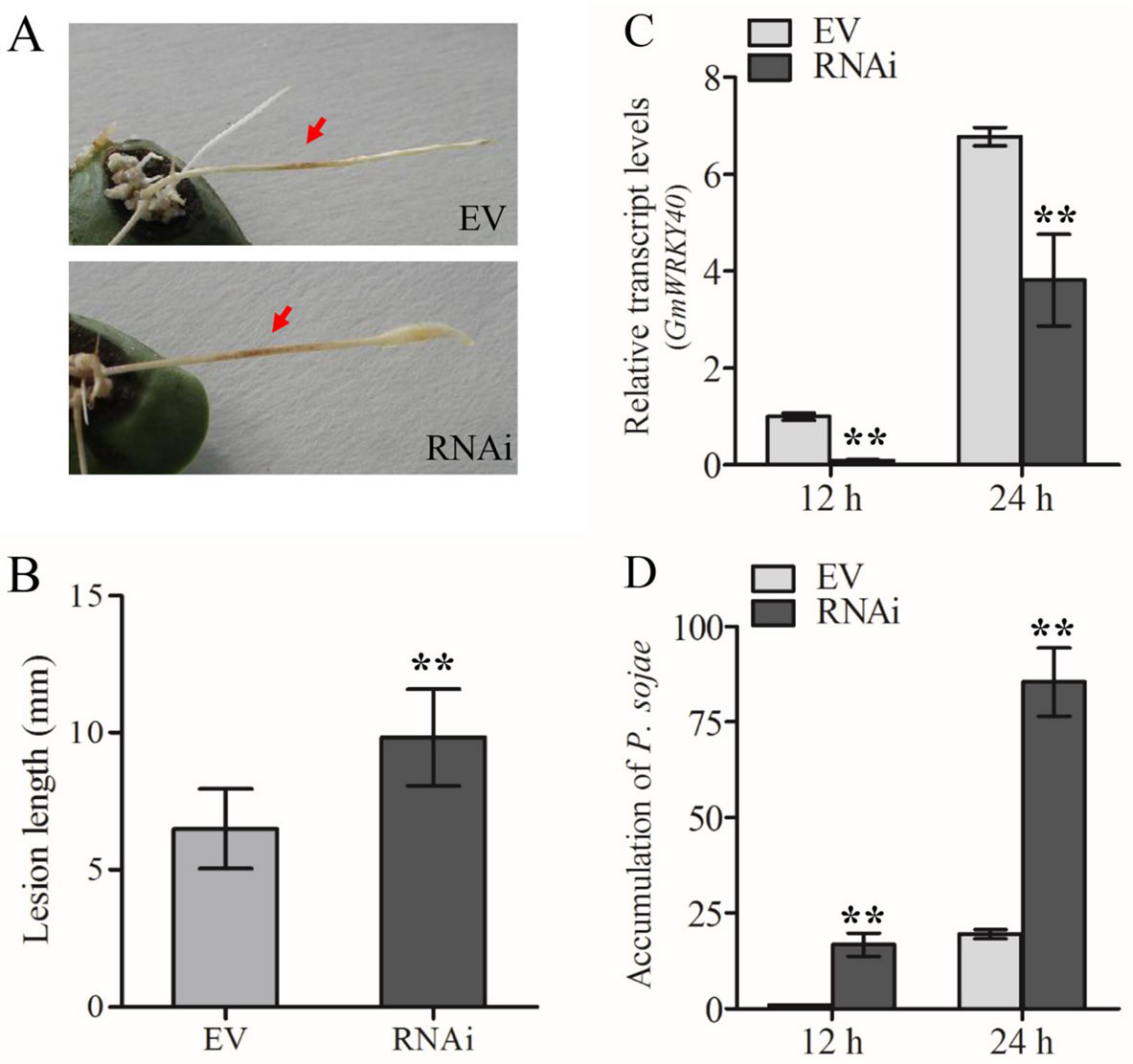
Transgenic soybean hairy roots with silenced GmWRKY40 exhibit enhanced susceptibility to *Phytophthora sojae*. **a** Disease phenotypes of *GmWRKY40*-RNAi Williams 82 hairy roots and control at 36 h after inoculated with P6497. **b** The lesion length of P6497 infected hairy roots. Lesion length was taken 36 hpi. **c** Relative expression of *GmWRKY40* was determined by qPCR at 12 and 24 hpi in *GmWRKY40* silencing or EV hairy roots (**d**) *P. sojae* biomass was determined by qPCR at 12 and 24 hpi in *GmWRKY40* silencing or EV hairy roots. The experiments all above were repeated three times along with at least three independent repetitions of the biological experiments. Error bars indicate the standard error. Asterisks indicate statistically significant differences (^**^P < 0.01)

To investigate whether *GmWRKY40* participate the resistance of soybean to *P. sojae*, transgenic roots expressing RNAi-*GmWRKY40* or RNAi empty vector were infected with *P. sojae*. The Williams 82 roots expressing RNAi empty vector exhibited slightly necrotic HR lesions, while infection of RNAi-*GmWRKY40* roots led to a more seriously extended lesions (Fig. 4a-b).

**Fig. 5:**
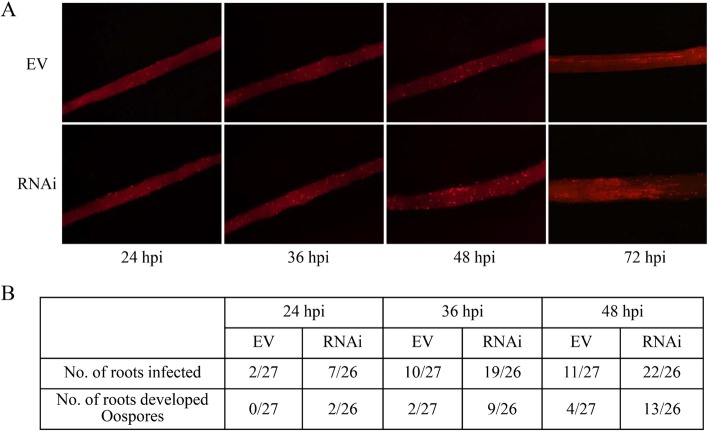
GmWRKY40 regulates soybean defense against *P. sojae* infection. **a** *GmWRKY40* silenced hairy roots were hyper-susceptible to *P. sojae* infection. Hairy roots were inoculated with zoospore suspension (10^4^ zoospore/mL) of the *P. sojae* P6497R. *P. sojae* infection was monitored using a fluorescent microscope at 24, 36 and 48 hpi. Bar equals 0.5 mm. **b** Infection progress of *P. sojae* in soybean roots expressing EV or RNAi-*GmWRKY40*. The number of roots allowing hyphae penetration or oospore development were counted at 24, 36 and 48 hpi. This experiment were repeated three times with similar results

To further analyze the influence of *GmWRKY40* to disease resistance in soybean, transgenic roots were inoculated with zoospore suspensions of *P. sojae* strain P6497R, which constitutively expresses red fluorescence protein (RFP). The number of roots that supported hyphae invading into root epidermis and oospore germination and extension at the inoculated area were counted at 24, 36 and 48 h post-inoculation, according to Wong et al. [57]. The obviously greater number of inoculated RNAi-*GmWRKY40* roots allowed hyphal invasion and oospore development compared to roots that expressing RNAi empty vector indicating that the RNAi-*GmWRKY40* roots exhibited hyper-susceptibility to *P. sojae* (Fig. 5). We also determined the *P. sojae* biomass accumulation by quantitative real-time PCR using primers specific to highly conserved repetitive sequences in the *P. sojae* genome. RNAi-*GmWRKY40* transgenic roots showed a higher growth rate of *P. sojae* biomass compared with RNAi empty vector (Fig. 4c-d). These results indicated that *GmWRKY40* as a positive regulator plays pivotal roles in soybean resistant to *P. sojae*.

### Suppression of *GmWRKY40* decreased the accumulation of ROS

**Fig. 6:**
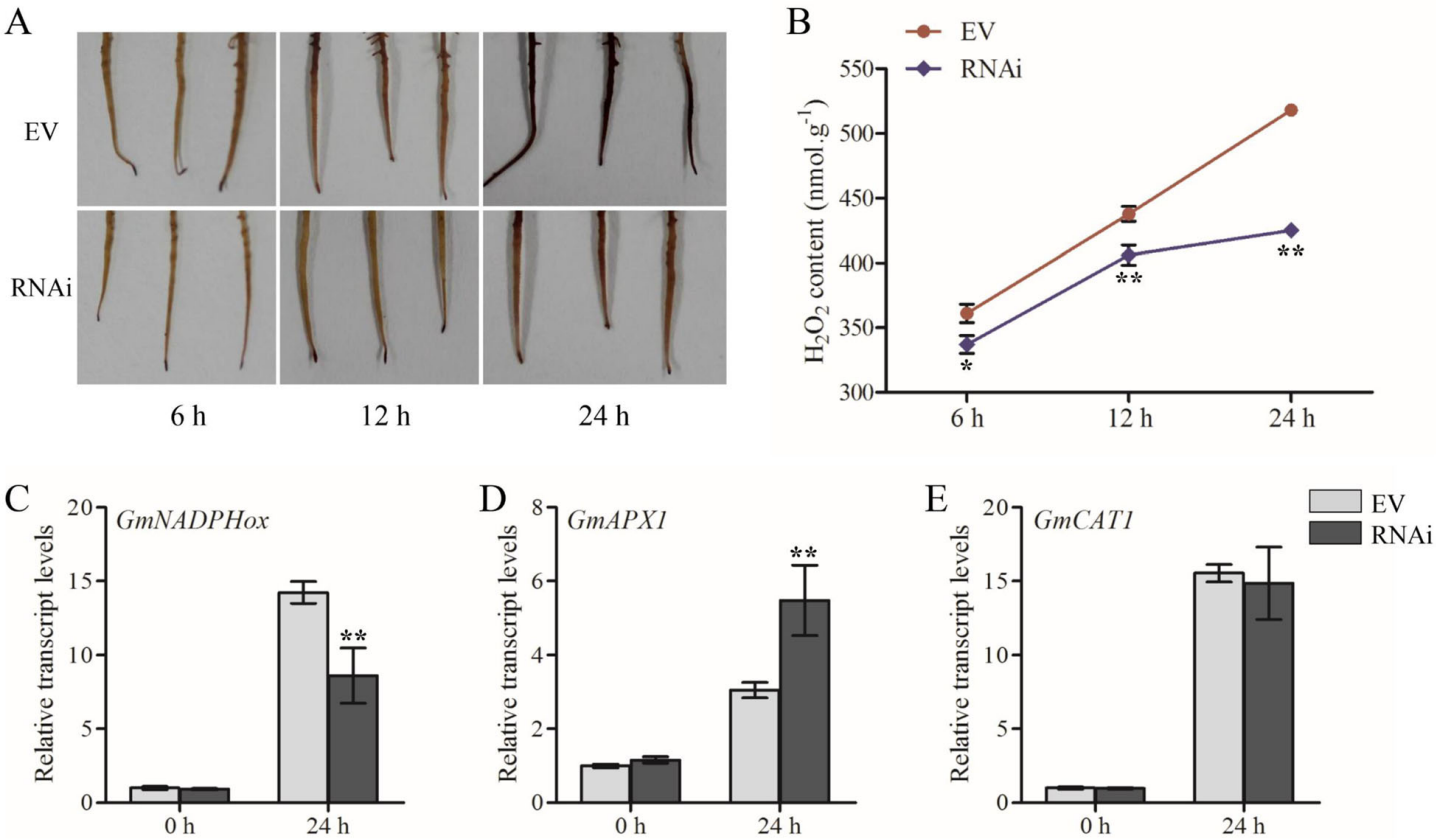
Silencing GmWRKY40 decreased the accumulation of ROS following *P. sojae* infection. **a** Accumulation of H_2_O_2_ was detected by DAB staining at 6, 12 and 24 h after inoculated with *P. sojae*. This experiment was repeated three times with similar results. **b** H_2_O_2_ content was measured at 6, 12 and 24 h after inoculated with *P. sojae*. Values represent the means of 3 replicates ± SD. Asterisks indicate statistically significant differences (*P < 0.05 or **P < 0.01). **c**-**e** Transgenic hairy roots were inoculated with zoospore suspension of *P. sojae* and samples were collected at 0 or 24 h after inoculation. Gene expression of *GmNADPHox*, *GmAPX1* and *GmCAT1* were analyzed by qRT-PCR. Values represent the means of 3 replicates ± SD. ^**^ Student’s *t*-test: *p* < 0.01

Reactive oxygen species (ROS) play vital roles in plant defenses as signaling molecules [58]. To check whether ROS accumulation was relevant to the hyper-susceptibility to *P. sojae* of the soybean, we detected the accumulation of H_2_O_2_ in RNAi-*GmWRKY40* transgenic roots and RNAi empty vector roots following exposure to *P. sojae*, showing that significantly decreased H_2_O_2_ production was detected in RNAi-*GmWRKY40* roots relative to RNAi empty vector roots (Fig. 6b). The 3, 3ˊ-diaminobenzidine (DAB) staining on the RNAi-*GmWRKY40* transgenic roots following *P. sojae* infection exhibited less brown relative to empty vector roots (Fig. 6a), which was consistent with the results measured above.

To explore the underlying mechanism of the reduced H_2_O_2_ levels in the transgenic roots during disease resistance process, the expression of oxidation-related genes in RNAi-*GmWRKY40* transgenic and control roots were analyzed in response to *P. sojae*. As shown in Fig. 6c-e, the transcript levels of *NADPHox* (NADPH oxidase, H_2_O_2_ synthesis-related gene) [59], *APX1* (ascorbate peroxidase 1) and *CAT1* (catalase 1) (ROS scavenging-associated genes) [60, 61] were not significantly different in RNAi-*GmWRKY40* transgenic roots before *P. sojae* infection compared with control roots, showing that *GmWRKY40* did not participate in the accumulation of H_2_O_2_ under normal conditions. After inoculation, the expression of *GmNADPHox* in RNAi-*GmWRKY40* transgenic roots was significantly lower than that in the control roots, otherwise, the expression of *GmAPX1* related to H_2_O_2_ scavenging was significantly up-regulated, indicating that *GmWRKY40* participated in the accumulation of H_2_O_2_ during the *P. sojae* infection by activating the H_2_O_2_ synthesis pathway and inhibition of H_2_O_2_ scavenging.

### GmWRKY40 interact with JAZ proteins

**Fig. 7:**
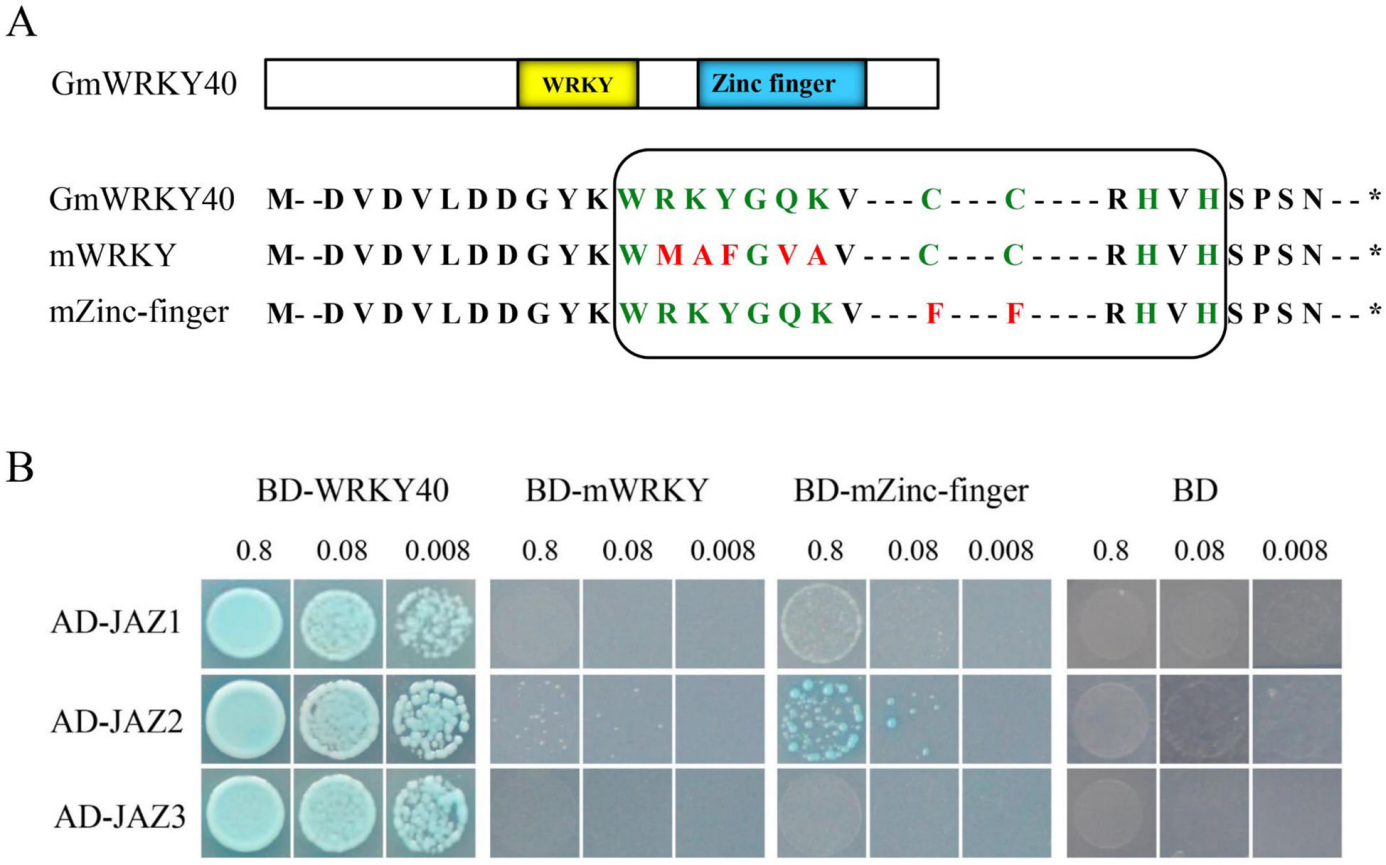
GmWRKY40 interacts with JAZ proteins. **a** Diagram of the GmWRKY40 protein functional domains and schematic of the amino acid mutation of WRKYGQK domain or Zinc finger domain. mWRKY indicate WRKY domain mutation; mZinc-finger indicate Zinc-finger domain mutation. **b** Yeast-two-hybrid assays. The Gal4 DNA binding domain was fused with GmWRKY40 (shown as BD-WRKY40, BD-mWRKY or BD-mZinc-finger) and the Gal4 activation domain was fused with JAZ1, JAZ2 or JAZ3 (shown as AD-JAZ1, AD-JAZ2 or AD-JAZ3). The Gal4 DNA binding domain expressed by pGBKT7 was used as a negative control. 0.8, 0.08, and 0.008 are the bacteria diluted concentration gradient

Increasing evidences suggested that WRKY proteins function by forming protein complexes with other interactors [33]. To determine which protein is responsible for the interaction with GmWRKY40, we performed yeast two-hybrid screening to identify GmWRKY40-interacting partners. There are three JAZ proteins (JAZ1-Glyma.07G041400.1, JAZ2-Glyma.01G204400.1, and JAZ3-Glyma.09G071600.1) represented among the candidate interactors (Here we temporarily designated JAZ1-JAZ8, including the 5 JAZ proteins mentioned below). To confirm their interaction in yeast, open-reading frame sequences of the eight proteins were fused with the activation domain (AD) of the pGADT7 vector and used for further interaction experiments with GmWRKY40. As shown in Fig. 7b, GmWRKY40 interacted strongly with the three JAZ proteins: JAZ1, JAZ2, and JAZ3. To further examine which domain of GmWRKY40 proteins is critical for the interactions with JAZ1, JAZ2, or JAZ3, pGBKT7 vectors containing a WRKY domain mutant or a zinc-finger domain mutant were used (Fig. 7a). The results showed that JAZ2 exhibited weak interaction with zinc-finger domain mutant protein, JAZ1 and JAZ3 exhibited no interactions neither with WRKY domain mutant protein nor with zinc-finger domain mutant protein (Fig. 7b).

**Fig. 8:**
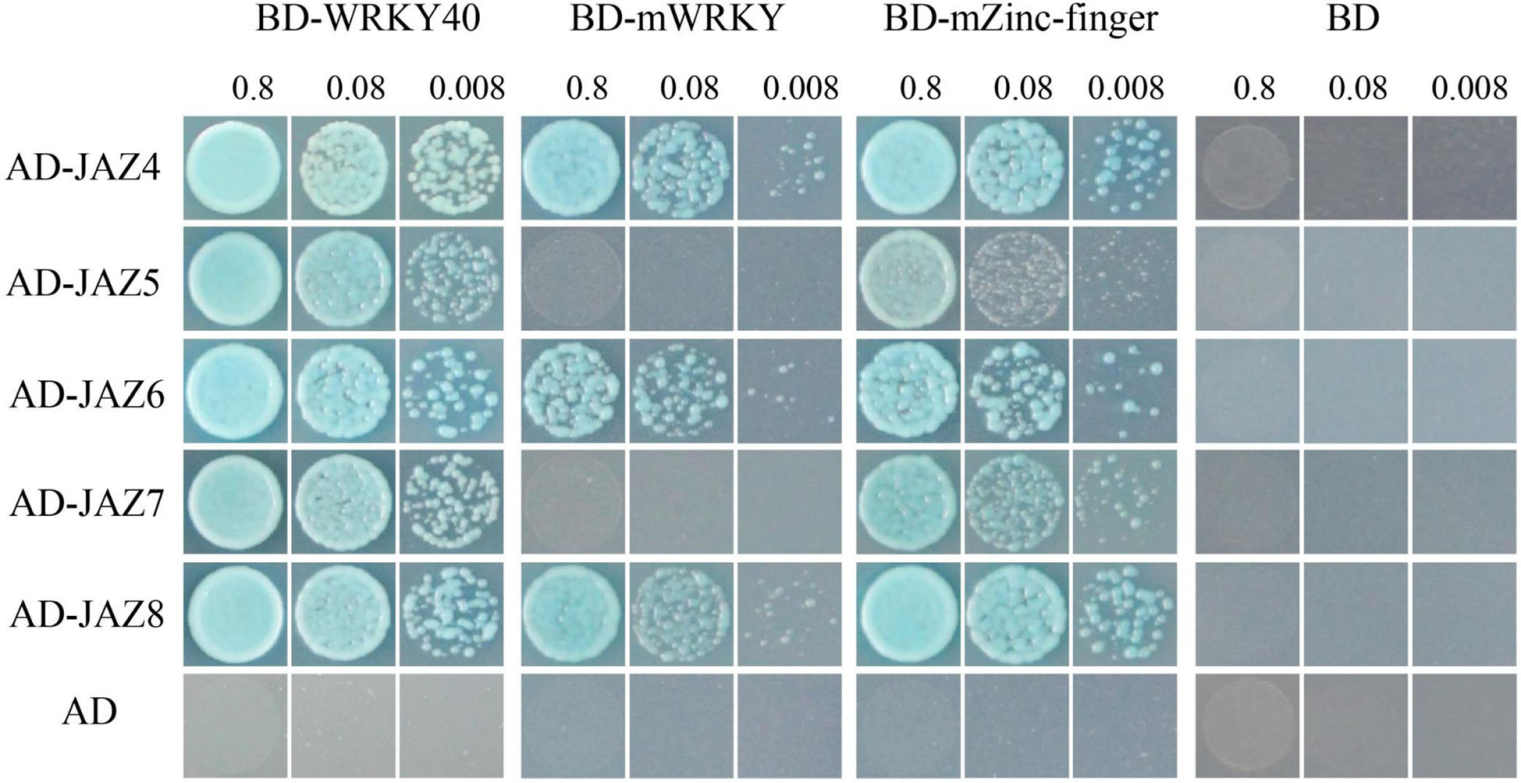
GmWRKY40 interacts with multiple JAZ family proteins. Yeast-two-hybrid assays. The Gal4 DNA binding domain was fused with GmWRKY40 (shown as BD-WRKY40, BD-mWRKY or BD-mZinc-finger) and the Gal4 activation domain was fused with JAZ4-JAZ8 (shown as AD-JAZ4 to AD-JAZ8). The Gal4 DNA binding domain expressed by pGBKT7 was used as a negative control. 0.8, 0.08, and 0.008 are the bacteria diluted concentration gradient

To gain further insights into the potential mechanisms of the reduced resistance in *GmWRKY40*-RNAi transgenic soybean roots, the gene expression between empty vector control and transgenic roots inoculated with *P. sojae* for 12 h was determined and compared using RNA sequencing. We detected multiple differentially expressed stress-relevant genes, including *JAZ* genes, *ERF* genes, peroxidase genes, transcription factors, and disease resistance-responsive genes (Additional file 2: Table S1). It was noteworthy that 8 JAZ proteins (Table 1) were induced in *GmWRKY40*-RNAi transgenic soybean roots after infected with *P. sojae*, including the previously tested three proteins interacted with GmWRKY40 (Fig. 7b). We further studied the interaction of other 5 JAZ proteins with GmWRKY40, with the result that they are interacted with GmWRKY40 either (Fig. 8). The interaction results of 5 JAZ proteins with domain mutated GmWRKY40 were shown in Fig. 8. WRKY domain of GmWRKY40 is necessary for interaction with JAZ5 (Glyma.16G010000.1) and JAZ7 (Glyma.15G179600.1). JAZ4 (Glyma.13G112000.1), JAZ6 (Glyma.11G038600.1), and JAZ8 (Glyma.17G047700.1) exhibited interact either with WRKY domain mutant protein or with zinc-finger domain mutant protein. The results suggested that there has different interaction patterns when GmWRKY40 interact with different JAZ proteins.

**Table 1:** RNA sequencing analysis of *JAZ* genes up-regulated in *GmWRKY40*-RNAi transgenic roots compared with EV soybean roots

	Accession	Gene product	Pathway	log2 Fold (RNAi/ EV)
JAZ1	Glyma.07G041400.1			1.848557332
JAZ2	Glyma.01G204400.1			3.400343215
JAZ3	Glyma.09G071600.1			1.593843195
JAZ4	Glyma.13G112000.1	jasmonate ZIM domain- containing protein	Hormone metabolism	1.575410461
JAZ5	Glyma.16G010000.1		Jasmonate signal transduction	1.987472879
JAZ6	Glyma.11G038600.1			3.403597988
JAZ7	Glyma.15G179600.1			1.444590913
JAZ8	Glyma.17G047700.1			2.415031607

## Discussion

In the plants response to various stresses, some transcription factors were activated and then subsequent a numerous defense-associated genes were transcriptional regulated, these changes are essential for the defense mechanisms establish. Genes respond with coordinated changes in transcriptional expression when the plant exposure to pathogen attack, then led to cellular and physiological reprogramming and the resistance to pathogen infection increasing [62]. Plenty of genes expression changes are regulated by various TFs. A large body of evidences suggests that WRKY TFs play important roles as positive or negative regulators in plant response to pathogen infection [33].

In this study, we performed a functional analysis of the soybean *WRKY40* gene, which was demonstrated to increase soybean resistance to *P. sojae*. It was reported that WRKY transcription factors response to biotic stresses in many plant species, including *Arabidopsis* [42, 63], rice [64, 65], barley [66], cotton [67], pepper [40], and tobacco [47, 68], among others. To our knowledge, a few WRKY transcription factors have been functionally demonstrated in soybean-pathogen interactions. Using virus-induced gene silencing, Pandey AK et al. identified 3 WRKY genes (*GmWRKY36*, *GmWRKY40*, and *WRKY45*) that play a role in *Rpp2*-mediated resistance toward *Phakopsora pachyrhizi* [69]. A genome-wide annotation of the soybean WRKY family was carried out and 75 genes were identified as involved in the soybean response to *P. pachyrhizi* infection based on transcriptional regulation [38]. *GmWRKY30* and *GmWRKY6* play a role in soybean *Rsv1*-mediated extreme resistance to *Soybean mosaic virus* [70]. The function of WRKY transcription factor response to *P. sojae* in soybean was characterized as well. Overexpression and RNA interference analysis demonstrated that *GmWRKY31* enhanced resistance to *P. sojae* [54]. Lin et al. performed comparative transcriptomic analysis to characterize genes and multiple branches of putative regulatory networks associated with resistance to *P. sojae* in 10 soybean NILs, it is apparent that some of the genes belonging to WRKY family play pivotal roles in defense signaling transduction [71]. Our investigation showed that *GmWRKY40* is induced by *P. sojae* infection (Fig. 3a), indicating its potential participation in modulating *P. sojae*. The transgenic roots, which presented decreasing transcripts of *GmWRKY40*, exhibited hyper-susceptibility to *P. sojae* (Figs. 4 and 5). In this experiment, we also used Williams (lacks any known *Rps* gene) to examine whether the *GmWRKY40* involved in basal resistance to *P. sojae*. The results showed that more serious extended lesions and greater *P. sojae* biomass accumulation were determined in RNAi-*GmWRKY40* roots compared with RNAi empty vector roots after inoculated *P. sojae* (Additional file 1: Figure S2). The results show that *GmWRKY40* is required for soybean response to *P. sojae*. Moreover, the research about soybean bHLH transcription factor GmPIB1 revealed that overexpression transgenic Williams (*rps*) hairy roots enhanced the resistance to *P. sojae*, while *GmPIB1*-RNAi transgenic ‘L77–1863’ (*Rps*1b) hairy roots exhibited more susceptibility [25]. In rice, Overexpression of *OsWRKY62* reduces the basal defense and *Xa21*-mediated resistance against *Xoo*, imply that OsWRKY62 functions as a negative regulator to *Xoo* in rice [72]. Here, our results demonstrated that *GmWRKY40* acted as a positive regulator in the defense response of soybean to *P. sojae*.

The phytohormones JA, SA, ET, and ABA are important signaling molecules involved in the plant response to pathogen infection [73]. WRKY transcription factor has been revealed function as key components in various hormone signaling pathways [42, 74]. For example, *WRKY70* acts as an activator of SA-induced genes and a repressor of JA-responsive genes, indicating that *WRKY70* is an important node of convergence integrating SA and JA signaling during plant defense responses [41]. *AtWRKY8* is involved in the defense response against TMV-cg through the direct regulation of *ABI4*, *ACS6*, and *ERF104* and mediates the crosstalk between ABA and ethylene signaling during the interaction between TMV-cg and *Arabidopsis* [75]. Our present study demonstrated that levels of *GmWRKY40* mRNA transcripts were significantly induced by *P. sojae* and diverse hormone signaling molecules stresses (Fig. 3). In addition, we analyzed the expression levels of SA signaling pathway associated genes, including *GmNPR1*, *GmPR1a* and *GmPR5*. It is reveal that *GmWRKY40* silencing led to the remarkably decreased *GmNPR1*, *GmPR1a* and *GmPR5* mRNA levels compared with the control after inoculated with *P. sojae* (Additional file 1: Figure S3). We deduce that *GmWRKY40* might be involved in soybean defense responses to *P. sojae* infection through phytohormones-mediated signaling pathways.

ROS network plays an important role in signal transduction of plant resistance to pathogens [58, 76]. ROS was rapidly produced following plant infected with pathogen, then preventing pathogen from entering the cell or the resistant genes were induced to inhibit pathogen growth [77, 78]. In plant-pathogen interactions, WRKYs played essential roles either in ROS bursts or in ROS scavenging. Previous work showed that WRKYs phosphorylated by MAPK was required for plant immune ROS bursts by activation of NADPH oxidase in *N. benthamiana* [47]. In our investigation, the lower H_2_O_2_ accumulation was observed in the transgenic roots of RNAi-*GmWRKY40* compared with control roots after inoculated with *P. sojae* (Fig. 6a-b). The transcript level of *GmNADPHox*, which associated with H_2_O_2_ synthesis [79], was detected lower in the RNAi-*GmWRKY40* transgenic roots relative to the control roots (Fig. 6c). We reasoned that silencing *GmWRKY40* down-regulated the expression of *GmNADPHox* resulting in reduced H_2_O_2_ accumulation and compromised transgenic soybean roots resistant to *P. sojae*.

JAZ proteins were identified as the transcriptional repressors of JA signaling [80]. Jasmonate treatment causes JAZ degradation and this degradation is dependent on activities of the SCF^COI1^ ubiquitin ligase and the 26S proteasome [81], leading to activation of various transcription factors, which subsequently regulate their downstream signal cascades and modulate respective plant responses [82, 83]. Some TFs have been reported as targets of JAZ proteins, such as bHLH TFs, MYB TFs and DELLAs [84]. In this study, we found that GmWRKY40 interacted with several JAZ proteins (Figs. 7 and 8), which are up-regulated in *GmWRKY40*-RNAi transgenic soybean roots after infected with *P. sojae* (Table 1). JAZ protein has been reported play roles in pathogen defense [83, 85]. Here we hypothesized that the up-regulation of JAZ proteins in *GmWRKY40* silenced transgenic roots enhances susceptibility to *P. sojae* infection by repression of the JA signaling. More work is required to understand the roles of these JAZ proteins in soybean response to pathogens. In addition, WRKY TFs also act on transcriptional level of *JAZ* genes expression. Jiang and Yu’s research showed that WRKY57 acts as transcriptional activator that binds to the promoter sequences of *JAZ1* and *JAZ5* to compete with the transcription regulation function of WRKY33, coordinate regulate Arabidopsis against *B. cinerea* [86]. We also tested which domain of GmWRKY40 responsible for the interaction with JAZ proteins. WRKY domain of GmWRKY40 is necessary for JAZ2/5/7-GmWRKY40 interaction, however JAZ4/6/8 exhibited interact both with WRKY domain mutant and zinc-finger domain mutated protein. JAZ1/3 had no interactions neither with WRKY domain mutant protein nor with zinc-finger domain mutant protein (Figs. 7 and 8). Thus, the WRKY domain and zinc-finger domain of GmWRKY40 protein was needed differently for the interaction with different JAZ proteins. The function of these JAZ proteins in soybean response to *P. sojae* and the mechanisms of diverse JAZ proteins interact with different domain mutated GmWRKY40 will be addressed in future studies to elucidate the underlying of GmWRKY40-mediated regulation in the soybean defense response.

## Conclusions

Collectively, the data presented in this study not only reveal an important regulatory function for *GmWRKY40* in soybean resistance to *P. sojae*, but also provide a foundation for further investigation of *P. sojae*-induced signaling pathways in which *GmWRKY40* participates. Our findings indicate that GmWRKY40 functions as a positive regulator in soybean plants response to *P. sojae* through modulating hydrogen peroxide accumulation or maybe interact with JAZ proteins. However, functional investigation of *GmJAZ* overexpression/ RNAi transgenic plants is necessary to further determine the functions of soybean *GmWRKY40* in biotic stress resistance. Moreover, further explorations on the identification of *GmWRKY40*-regulated downstream genes will be useful to clarify the mechanisms of *GmWRKY40* in the regulation of soybean response to *P. sojae*. These findings will expand our understanding towards the function of *GmWRKY40* and will provide invaluable resources for understanding the molecular interaction between soybean and *P. sojae*.

## Materials and methods

### Plant materials, growth conditions and treatments

Soybean (*Glycine. max*) seeds of Williams 82 were grown in greenhouse at 25 °C under a 16-h-light and 8-h-dark photoperiod. Seven-days-old seedlings were infected with *P. sojae* isolate P6497 by hypocotyl inoculation method [87, 88]. Samples were harvested at 0, 3, 6, 12, 24, 36, 48 and 72 h post-inoculation (hpi), frozen directly in liquid nitrogen and then stored at − 80 °C for later use. The soybean seeds and *P. sojae* used in this study provided in the Laboratory of National Center for Soybean Improvement of Nanjing Agricultural University.

For hairy root induction, soybean seeds were surface-sterilized by chlorine gas for 1 h, soaked overnight in sterilized water and then germinated on agar (0.8%) medium and grown in a growth chamber (25 °C, 16 h photoperiod) for 6 days. Cotyledons were then collected for *Agrobacterium rhizogenes*-mediated hairy roots transformation.

For phytohormone treatment, soybean seeds were planted into vermiculite 7 days, then roots were cleaned and submerged in different hormones with the concentration of 100 μM methyl jasmonate (MeJA), 2 mM ethylene (ETH), 5 mM salicylic acid (SA) and 50 mM abscisic acid (ABA). Roots were harvested at 0, 1, 3, 6, 12 and 24 h, frozen in liquid nitrogen immediately and stored at − 80 °C until RNA extraction.

*Nicotiana benthamiana* plants were grown at 25 °C with a 16 h photoperiod in greenhouse. Plants of 5–6 weeks old were used for agroinfiltration.

### *Phytophthora sojae* culture conditions

*P. sojae* isolate P6497 was cultured at 25 °C on 1.5% V8 juice agar medium. V8 medium containing 40 μg mL^− 1^ geneticin were used to culture the RFP-labeled strain P6497R [89]. For zoospore preparation, cultivate the newly cultured P6497R mycelium in V8 liquid medium, then incubated at 25 °C in dark for 2 days. The mycelium was then washed several times with sterile ddH_2_O and incubated at 25 °C in the dark for about 12 h to release zoospores [90]. Finally, the released zoospores were collected to inoculate hairy roots of soybean.

### Vectors construction

To generate *GmWRKY40* RNAi construction, the 302 bp cDNA specific fragment of *GmWRKY40* gene was amplified by PCR using primers of P12-WRKY40-F and P12-WRKY40-R, and then cloned into pDONR221, a Gateway entry vector. Finally, through Gateway LR Clonase reaction, the *GmWRKY40* fragment was cloned into pHellsGate12: GFP [21]. The constructed vector was transformed into *Agrobacterium rhizogenes* K599 strain for further transformation.

For yeast-two-hybrid analysis, the full-length *GmWRKY40* cDNA was amplified by PCR using primers of WRKY40-BD-F and WRKY40-BD-R, and then cloned into the bait vector pGBKT7 and then transformed into the yeast strain AH109. The full-length JAZ1-JAZ8 coding sequences (CDSs) were cloned into the prey vector pGADT7 and then transformed into the yeast strain Y187. The primers used for yeast two-hybrid are listed in Additional file 3: Table S2.

### Subcellular localization of GmWRKY40

The full coding sequence of *GmWRKY40* without termination codon was amplified by primers WRKY40-SCL-F and WRKY40-SCL-R, with *Kpn* I and *Sma* I restriction sites respectively, and then constructed into the pBIN-GFP vector to generate *CaMV 35S: GmWRKY40-GFP* conctruct. The empty pBIN-GFP vector was used as a control. The both constructs were transformed into *Agrobacterium tumefaciens* strain GV3101 by electroporation. The validated individual clones were shaken in 4 ml LB broth (with 50 mg L^− 1^ kanamycin) for 24 h at 28 °C. Bacteria liquid were washed with 10 mM MgCl_2_ to a final OD_600_ of 0.5. The bacterial suspension was infiltrated into the abaxial side of fully expanded 5–6 weeks old tobacco leaves. After infiltration, the plant were kept in the greenhouse 48 h for inoculation, and then the tissue was strained with DNA-specific nuclear strain 4′, 6-diamidino-2-phenylindole (DAPI) for 10 min. The GFP signals were detected by a confocal fluorescence microscope (Zeiss, Germany).

### *Agrobacterium* culture and soybean Cotyledon transformation

The plasmid vectors used for silencing of *GmWRKY40* and the empty vector pHellsGate12: GFP were transformed into *A. rhizogenes* K599 strain by electroporation. LB media with 50 μg·mL^− 1^ kanamycin were used for select the positive clones, and colony PCR was performed to validate the individual clones. The single-positive colony was shaken in LB broth containing 50 μg·mL^− 1^ kanamycin at 28 °C for 24 h. The bacteria were centrifuged at 4500 rpm for several minutes and suspended in 10 mM MgCl_2_ (final OD_600_ is 0.5) for soybean cotyledon transformation to obtain soybean hairy roots.

Soybean hairy root transformation was performed according to Graham et al. [91] and Yan et al. [21].

### Assay of *P. sojae* infection of soybean hairy roots

For root inoculation, the transformed hairy roots of soybean were screened under microscope (OLYMPUS MVX10, Japan) by the GFP label in the vector. Then, the roots were inoculated with P6497 mycelia agar according to previous described by Graham et al. [91]. Venire caliper was used to measure the lesion length, and the significance of differences between the control roots and RNAi-*GmWRKY40* roots was determined by performing Student’s *t* test.

Detached hairy roots were immersed in the zoospore suspensions (approximately 10^4^ zoospore ml^− 1^) of *P. sojae* P6497R for 10 min. Inoculated roots were incubated in 0.6% agar plates in the dark at 25 °C for 48 h. Disease progression including hyphae extension and oospore development were monitored using a fluorescence microscope (OLYMPUS MVX10, Japan). *P. sojae* biomass was measured by qPCR using primers designed to amplify the *TEF 1* gene that is specific to *P. sojae* [21].

### Analysis of H_2_O_2_ accumulation

The H_2_O_2_ burst was assessed by staining infected soybean hairy roots with 3, 3′-diaminobenzidine (DAB, 1 mg/ml, pH 3.8) for 6–8 h at 25 °C in the dark. Representative phenotypes were photographed with camera.

The content of H_2_O_2_ was analyzed with a hydrogen peroxide assay kit (Beyotime, S0038) according to the manufacturer protocols. Samples to be tested were placed at room temperature for 30 min and measured immediately with a spectrometer at a wavelength of 560 nm. Absorbance values were calibrated to a standard curve generated with known concentrations of H_2_O_2_.

### Yeast two-hybrid screening and confirmation

Yeast transformation was performed by the LiAc and polyethylene glycol method, as described in the Yeast Protocols Handbook (Clontech, USA.). Yeast transformants were screened on synthetic dropout nutrient medium with X-α-gal but lacking leucine, tryptophan, histidine, and adenine. Blue colonies indicate a positive interaction between two proteins in yeast.

### RNA isolation and quantitative RT-PCR

Total RNA was extracted using the RNA simple total RNA kit (TIANGEN, China) according to the kit instructions, and total RNA samples were digested with DNase I to remove genomic DNA. RNA was then used to synthesize the first strand of cDNA using reverse transcriptase (TaKaRa, Japan) in accordance with the manufacturer’s protocol. Real-time PCR was performed using the cDNA and gene-specific primers (Additional file 3: Table S2). Each cDNA was amplified by quantitative PCR using the AceQ qPCR SYBR® Green Master Mix (Vazyme, China) with a Light Cycler® 480 (Roche, Germany). The soybean *GmCons 4* gene was used as internal control in reactions. The 2^-△△CT^ comparative CT method was used to estimate the relative expression level of genes. Students’ *t*-test was used for statistical analysis.

## Supplementary information

**Additional file 1: Figure S1.** GFP fluorescence in transgenic soybean hairy roots and analysis of silence efficiency. (A) Green fluorescence observed from a small portion of *Agrobacterium*-induced hairy roots. Roots were examined under a fluorescence microscope. (B) The expression of *GmWRKY40* were validated in each individual hairy root by quantitative RT-PCR. Error bars indicate the standard error. Asterisks indicate statistically significant differences (^**^P < 0.01). **Figure S2.** *GmWRKY40* participate in soybean basal resistance. (A) The lesion length after *P. sojae* infected with Williams hairy roots. Lesion length was taken at 24 hpi. (B) *P. sojae* biomass was determined by qPCR at 12 and 24 hpi in *GmWRKY40* silencing or EV Williams hairy roots. The experiments above were repeated three times along with at least three independent repetitions of the biological experiments. Error bars indicate the standard error. Asterisks indicate statistically significant differences (^**^P < 0.01). **Figure S3.** Expression patterns of SA signaling pathway genes in RNAi-*GmWRKY40* or EV hairy roots. Transgenic hairy roots were inoculated with zoospore suspension of *P. sojae* and samples were collected at 0 and 24 h after inoculation. Gene expression of *GmNPR1*, *GmPR1a* and *GmPR5* were analyzed by qRT-PCR. The experiments were repeated three times. Error bars indicate the standard error. Asterisks indicate statistically significant differences (**P < 0.01)

**Additional file 2: Table S1.** List of partial differentially expressed genes in RNAi-*GmWRKY40* transgenic roots after infection with *P. sojae* when compared to control roots

**Additional file 3: Table S2.** Primers used in this study

## Data Availability

The datasets used and/or analyzed during the current study available from the corresponding author on reasonable request.

## Abbreviations

ABA: Abscisic acid
APX1: Ascorbate peroxidase 1
CAT1: Catalase 1
DAB: 3, 3′-diaminobenzidine
DAPI: 4′, 6-diamidino-2-phenylindole
ETH: Ethylene
ETI: Effector-triggered immunity
ETS: Effector-triggered susceptibility
EV: Empty vector
JAZ: Jasmonate ZIM-domain
MeJA: Methyl jasmonate
NADPHox: NAD(P) H oxidase
PAMPs: Pathogen-associated molecular patterns
PRRs: Pattern recognition receptors
PTI: PAMP-triggered immunity
qRT-PCR: Quantitative reverse transcription PCR
ROS: Reactive oxygen species
SA: Salicylic acid
TFs: Transcription factors
