# Supplementary figures and images for: GmWRKY40, a member of the WRKY transcription factor genes identified from Glycine max L., enhanced the resistance to Phytophthora sojae
Source: BMC Plant Biol. 2019 Dec 30;19:598. doi: 10.1186/s12870-019-2132-0 (PMC6937711; doi:10.1186/s12870-019-2132-0)

## Slide 1
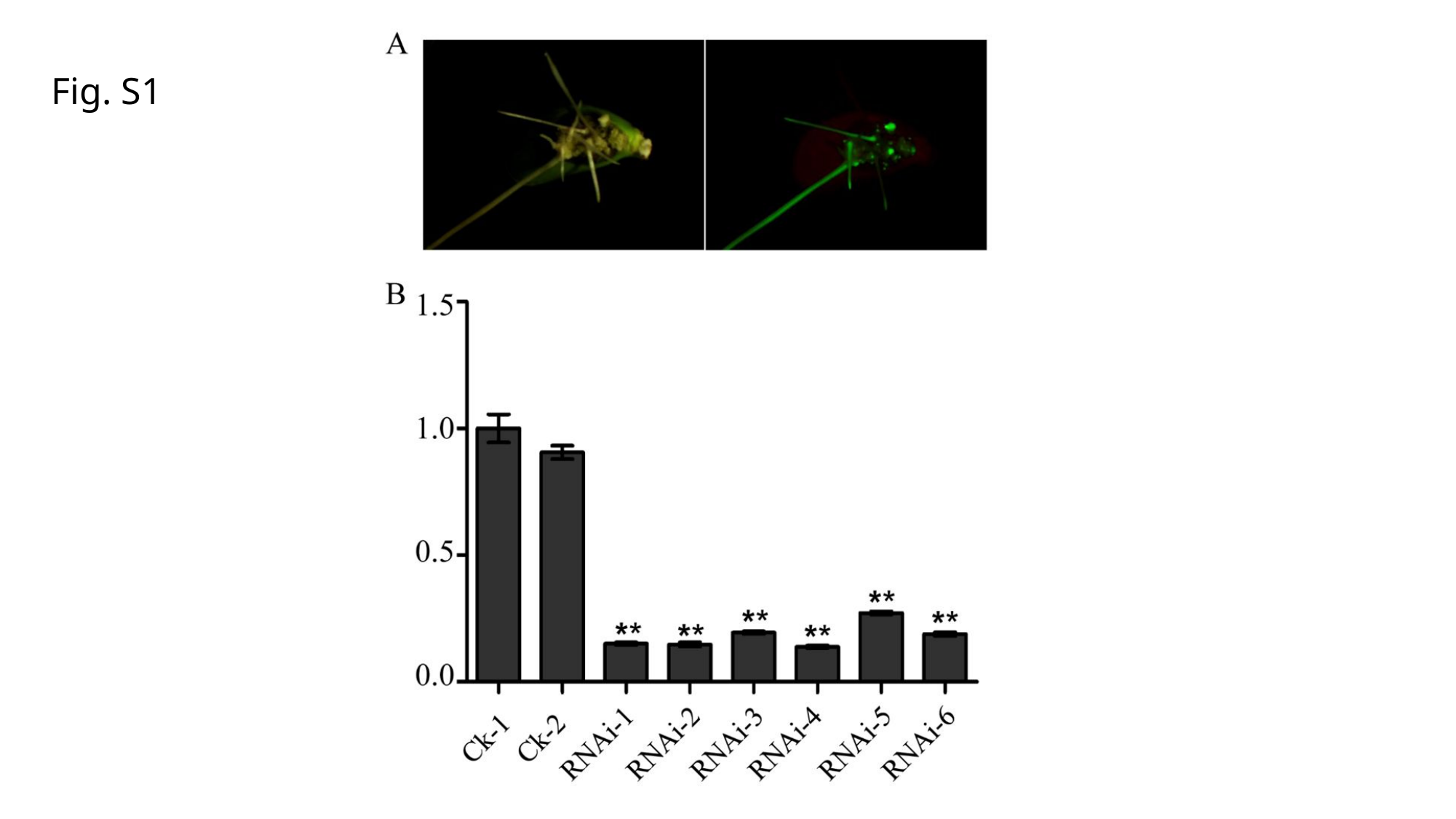

Fig. S1

## Slide 2
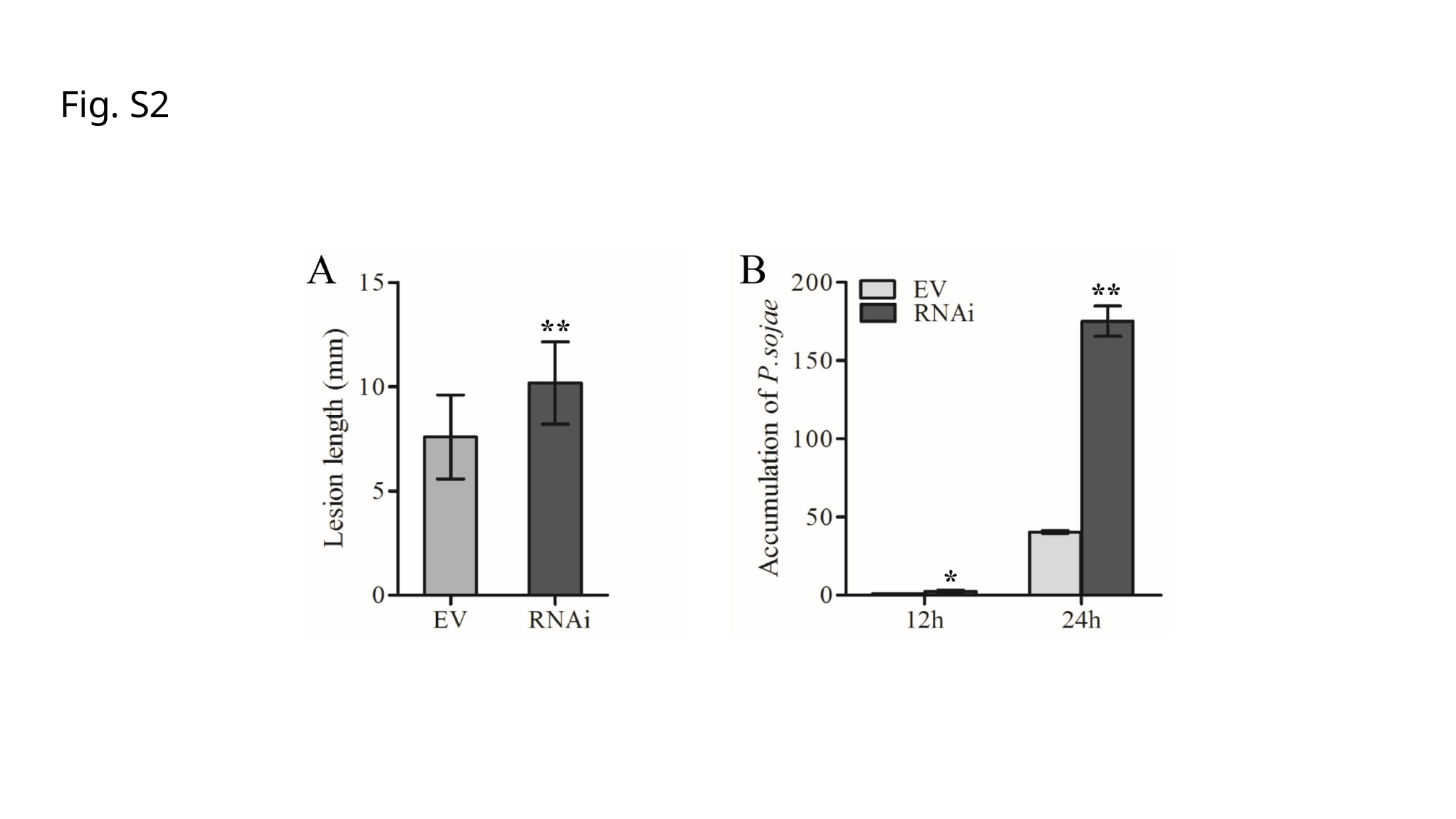

Fig. S2

## Slide 3
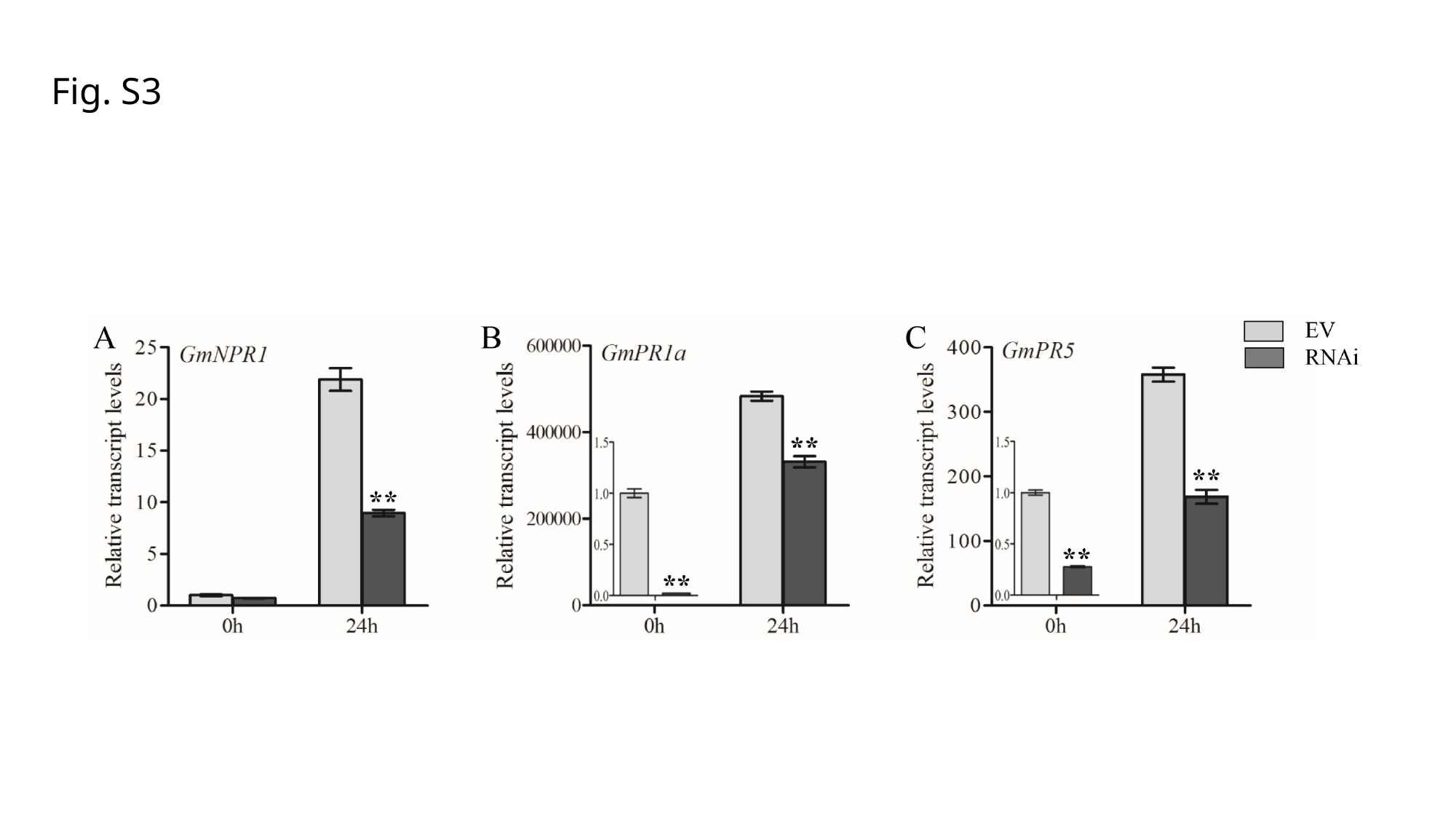

Fig. S3

Supplement: Supplementary file 1 — Additional file 1: Figure S1. GFP fluorescence in transgenic soybean hairy roots and analysis of silence efficiency. (A) Green fluorescence observed from a small portion of Agrobacterium-induced hairy roots. Roots were examined under a fluorescence microscope. (B) The expression of GmWRKY40 were validated in each individual hairy root by quantitative RT-PCR. Error bars indicate the standard error. Asterisks indicate statistically significant differences (**P < 0.01). Figure S2. GmWRKY40 participate in soybean basal resistance. (A) The lesion length after P. sojae infected with Williams hairy roots. Lesion length was taken at 24 hpi. (B) P. sojae biomass was determined by qPCR at 12 and 24 hpi in GmWRKY40 silencing or EV Williams hairy roots. The experiments above were repeated three times along with at least three independent repetitions of the biological experiments. Error bars indicate the standard error. Asterisks indicate statistically significant differences (**P < 0.01). Figure S3. Expression patterns of SA signaling pathway genes in RNAi-GmWRKY40 or EV hairy roots. Transgenic hairy roots were inoculated with zoospore suspension of P. sojae and samples were collected at 0 and 24 h after inoculation. Gene expression of GmNPR1, GmPR1a and GmPR5 were analyzed by qRT-PCR. The experiments were repeated three times. Error bars indicate the standard error. Asterisks indicate statistically significant differences (**P < 0.01) [file 12870_2019_2132_MOESM1_ESM.pptx]
